# Platelet Distribution Width Levels Can Be a Predictor in the Diagnosis of Persistent Organ Failure in Acute Pancreatitis

**DOI:** 10.1155/2017/8374215

**Published:** 2017-12-27

**Authors:** Feiyang Wang, Zibo Meng, Shoukang Li, Yushun Zhang, Heshui Wu

**Affiliations:** Department of Pancreatic Surgery, Union Hospital, Tongji Medical College, Huazhong University of Science and Technology, Wuhan 430022, China

## Abstract

**Purpose:**

The change of serum platelet indices such as platelet distribution width (PDW) has been reported in a series of inflammatory reaction and clinical diseases. However, the relationship between PDW and the incidence of persistent organ failure (POF) in acute pancreatitis (AP) has not been elucidated so far.

**Materials and Methods:**

A total of 135 patients with AP admitted within 72 hours from symptom onset of AP at our center between December 2014 and January 2016 were included in this retrospective study. Demographic parameters on admission, organ failure assessment, laboratory data, and in-hospital mortality were compared between patients with and without POF. Multivariable logistic regression analyses were utilized to evaluate the predictive value of serum PDW for POF.

**Results:**

30 patients were diagnosed with POF. Compared to patients without POF, patients with POF showed a significantly higher value of serum PDW on admission (14.88 ± 2.24 versus 17.60 ± 1.96%, *P* < 0.001). After multivariable analysis, high PDW level remained a risk factor for POF (odds ratio 39.42, 95% CI: 8.64–179.77; *P* < 0.001). A PDW value of 16.45% predicted POF with an area under the curve (AUC) of 0.870, a sensitivity with 0.867, and a specificity with 0.771, respectively.

**Conclusions:**

Our results indicate that serum PDW on admission could be a predictive factor in AP with POF and may serve as a potential prognostic factor.

## 1. Introduction

Acute pancreatitis (AP) is a common urgent abdominal condition with symptoms of upper abdominal pain, abdominal distention, nausea, and vomiting at onset. According to the revised 2012 Atlanta criteria, AP can be classified as mild (MAP), moderately severe (MSAP), and severity (SAP) through the occurrence of local/systemic complications and/or transient/persistent organ failure [1]. As the most serious complication in SAP, persistent organ failure (POF) can markedly increase the risk of death (36%–50%) compared with MSAP (<8%) [2]. So, early prediction of POF in AP is quite important for patients who need aggressive treatments. Study shows that early-stage fluid resuscitation, prophylactic antibiotics, nutritional support, and target organ therapy to SAP patients can reduce POF occurrence and mortality in hospital effectively [3]. It is also beneficial for MAP and MSAP patients who are not willing to have POF to reduce cost and unnecessary treatments. However, it is difficult to evaluate a patient on admission whether she will have POF, especially in the first 48 hours. So, we require a new predictive tool to judge the happening of POF.

In addition to the important role in hemostasis and thrombosis, platelet also contributes to the inflammatory process [4]. Several platelet indices are observed as diagnostic or prognostic factors in many infectious diseases [5]. Platelet distribution width (PDW) is a regular parameter in blood routine examination which reflects variation of platelet size distribution with a range from 8.3% to 56.6% [6, 7]. There is always a morphological change when platelet is activated in the environment of inflammation. Thus, PDW can be utilized as a sign of activated platelet releasing in some inflammatory diseases. Studies have demonstrated that PDW level changes under specific conditions compared to healthy individuals [8, 9].

We know that AP is a systemic inflammatory response involving multiple organs. Platelet undergoes a series of changes during inflammation and reflects this process through various platelet indices. Here, we analyzed whether PDW could be a predictive factor in AP patients who have POF. In addition, we compared it with the Rason and SIRS scoring systems and other clinical parameters.

## 2. Materials and Methods

### 2.1. Patients

We retrospectively analyzed the data from 135 patients admitted in the Pancreatic Surgery Department of Wuhan Union Hospital with confirmed diagnosis of AP from December 2014 to January 2016. The diagnosis criteria of AP are based on two or more of the following conditions: (1) upper abdominal pain of acute onset, (2) serum amylase or lipase activity three times greater than normal, and (3) finding on cross-sectional abdominal imaging consistent with AP [2]. To ensure the eligible patients, patients with chronic pancreatitis, pancreatic tumor, and admission in hospital longer than 72 hours from the onset of symptoms or incomplete laboratory examination results were excluded. All blood samples were collected within 2 hours after admission and analyzed within 6 hours in the same laboratory in Wuhan Union Hospital. Demographic and clinical characteristics were reviewed patients' electronic medical records and paper charts. The Wuhan Union Hospital ethics review board approved this study.

### 2.2. Definition and Etiologies

The severity of disease was determined by the revised Atlanta definition criteria 2012. Organ failure (OF) was defined by (1) PaO_2_/FiO_2_ < 300 mmHg; (2) serum creatinine > 170 *μ*mol/L; and (3) systolic blood pressure < 90 mmHg not responsive to fluid resuscitation [2]. Multiple organ failure (MOF) satisfied two or more conditions mentioned above and POF meant OF lasted more than 48 hours. Pancreas necrosis (PNec) was defined as pancreatic parenchymal and/or peripancreatic necrosis appearance on computed tomography (CT) images.

The etiologies of AP were generalized to be the following four classifications: (1) biliary, (2) alcohol, (3) hyperlipidemia, and (4) idiopathic. Biliary factor was considered if biliary tract stone was found through the detection of CT, ultrasound, magnetic resonance cholangiopancreatography (MRCP), endoscopic retrograde cholangiopancreatography (ERCP), or operation. If one drinks regularly at least 100 g/day or 250 g/week for more than 5 years, alcohol is the etiological factor of AP. For AP due to hyperlipidemia, plasma triglycerides or cholesterol level higher than 2.3 mmol/L and 5.17 mmol/L was necessary. If lack of any causes above or unexplained reasons, an idiopathic AP was considered.

### 2.3. Statistical Analysis

Statistical Production and Services Solution 19.0 (SPSS 19.0, SPSS Inc., Chicago IL, USA) was used in statistical analysis. Continuous data and categorical variables were presented as the means ± standard derivation (SD) and frequency, respectively. Baseline characteristics were compared between study and control groups by using Student's *t-*test. Categorical variable comparisons were performed by using the chi-square test. Univariable analysis used a log-rank test. All variables with statistically significant predictive value in univariable analysis were selected for further multivariable analysis. A Cox regression model was used in multivariable analysis. Odds ratios (ORs) and 95% confidence intervals (95% CIs) were presented. Receiver-operator curves (ROC) were presented to evaluate the sensitivity and specificity of the parameters in predicting POF in AP. *P* value < 0.05 showed a statistical difference.

## 3. Results

### 3.1. Patients

A total of 135 patients with confirmed AP admitted to the Union Hospital (Wuhan) from December 2014 to January 2016 were included in this study. The basic characteristic of high-PDW group (>16.45%, *n* = 50) and low-PDW group (≤16.45%, *n* = 85) was presented in Table 1. Compared with the low-PDW group, high-PDW group showed a notable higher incidence of POF (52.0% versus 4.7%, *P* < 0.001), PNec (52.0% versus 23.5%, *P* = 0.001), and in-hospital mortality (14.0% versus 4.7%, *P* < 0.001). Overall, 26 of these patients had solitary POF (all of respiratory system) and 4 patients were observed with multiple POF (all of respiratory + renal system) according to Table 2. At the same time, 22 patients with solitary POF (81.5%) and all patients with multiple POF developed PNec during hospitalization.

### 3.2. Comparison between Patients with and without POF

The clinical and the demographic characteristics of patients with and without POF were demonstrated in Table 3. The mean age of POF and non-POF patients was 46.7 ± 16.70 and 49.4 ± 14.01 years, respectively (*P* = 0.369). There was also no statistically significant difference between the gender, alcohol, smoke, and etiology of the groups.

**Figure 1:**
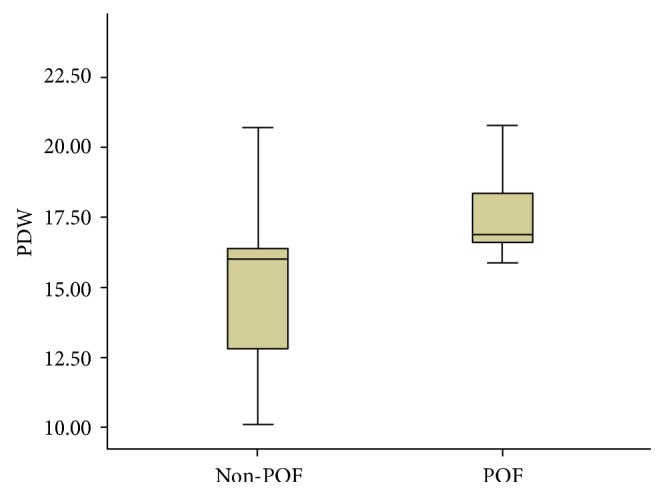
Box diagram of average serum PDW of POF and non-POF patients in AP.

Compared to patients without POF, the levels of hemoglobin, serum glucose, and PDW significantly increased in patients with POF, while the levels of albumin and serum calcium decreased (Table 4). Scoring systems like the Ranson scores and AP outcomes such as PNec and in-hospital mortality also showed a statistical difference (*P* < 0.05) between patients with and without POF (Table 4). Data about patients with POF having a higher average serum PDW level than non-POF one was shown in Figure 1.

### 3.3. Admission PDW Is an Independent Predictive Factor for POF

**Figure 2:**
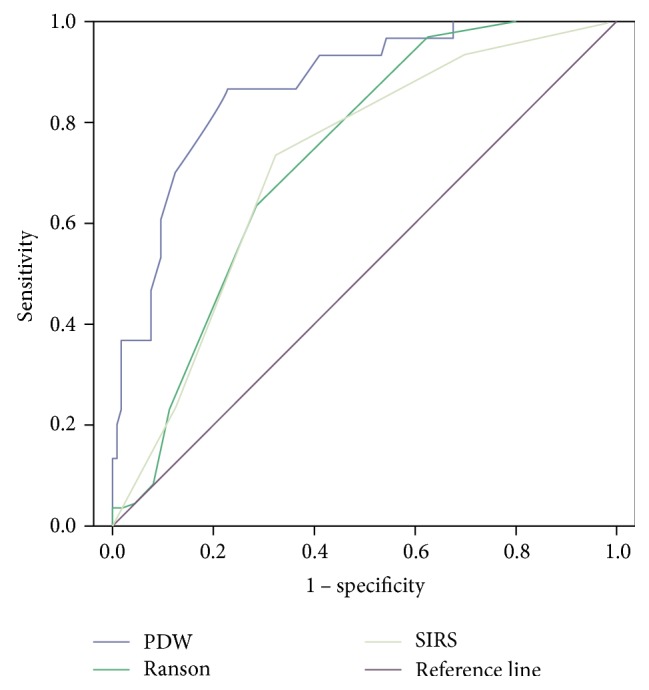
The ROC curve of PDW, Ranson, and SIRS scoring systems in diagnosing POF in AP.

In the univariable analysis, we observed that hemoglobin, serum glucose, albumin, serum calcium, PDW, and Ranson scores were significantly correlated with the incidence of POF. Then, these characteristics that showed statistical difference (*P* < 0.05) above were performed in multivariable analysis. The PDW level (>16.45%) could be a prognostic factor of POF (OR: 39.42, 95% CI: 8.46–179.77, *P* < 0.001) (Table 5). As shown in Table 6, serum PDW in admission had an area under the curve of the receiving operating characteristic (AUC) of 0.870 (95% CI: 0.801–0.939), with a sensitivity of 0.867 and a specificity of 0.771. The optimal threshold of PDW was 16.45% (Table 6 and Figure 2). These results showed that PDW had a better predictive value of POF in AP than the currently used Ranson and SIRS scoring systems.

## 4. Discussion

In our research, we found the correlation between the PDW and the incidence of POF in AP. Our result showed that PDW detected on admission was significantly higher in patients with POF than patients without POF. After multivariable analysis, PDW was classified as an independent predictive factor for POF. Therefore, PDW could be a relevant risk factor of the occurrence of POF which was superior to some traditional inflammation biomarkers. According to Seker et al.'s report [10], ESR may be influenced by the age and gender of patients and the presence of noninflammatory events such as anemia and renal failure, while CRP level begins to rise 48 hours after the onset of symptoms which has similar limitation to ESR.

As a common biomarker in platelet indices, PDW can act as an indicator of platelet volume variability and increases in the presence of platelet anisocytosis [11]. In addition, PDW can be affected by the morphology change of platelet when the platelet is activated during inflammation and thrombosis process [12]. Meanwhile, relationship between serum PDW value and some inflammatory or clinical disorders has been evaluated by Sahin et al. They found that PDW played an important role in identifying pulmonary tuberculosis, coronary artery disease, Alzheimer's disease, and acute cholecystitis [13–16]. In Bulent et al.'s study with 295 acute appendicitis patients, they demonstrated that PDW was an independent diagnosis criterion superior to white blood cell count and neutrophil percentage in acute appendicitis [12]. Bae et al. also revealed that PDW combined with white blood cell count and hemoglobin could be a short-term prognostic marker in patients with acute myocardial infarction because of the thromboembolic events [17]. Similarly, in a canine model of endotoxemia, PDW increased in septic subjects compared to controls. The author proposed to use platelet indices in the diagnosis and monitoring of endotoxemia [18]. These findings mentioned above suggest that significantly mutative level of PDW is indicative of some disease conditions. However, to the best of our knowledge, there is no report on PDW regarding the diagnosis of POF in AP. So we firstly evaluate PDW as a marker of POF in AP.

The value of PDW is usually calculated by a function of standard deviation of log platelet volume. PDW can directly measure variability in platelet size and reflect the heterogeneity in platelet morphology [19–21]. Reports show that platelet participates in the proinflammatory process by releasing proteins and small molecules from their granules, which can influence the function of the vascular wall and circulating immune cells [4, 22–24]. During inflammation, numerous inflammatory mediators like interleukin-1 participate in the process of platelet activation at the same time [25]. Activation of platelet causes morphologic change, including both shapes changing from discoid to spherical and pseudopodia formation. Progressive activated platelet with pseudopodia formation can have heterogeneous size, performing larger PDW value [16]. So the relationship between platelet and inflammation is mutual. Platelet promotes inflammatory progress and inflammation changes platelet shape. As a result, PDW can reflect an inflammatory condition in some infectious diseases as a platelet marker. Systemic inflammation response, which is frequently observed in SAP, may contribute to the release of inflammatory mediators, resulting in high level of PDW by the change of platelet morphology. Meanwhile, inflammatory mediators and cytokines released by platelet increase the permeability of vascular wall and lead to fluid extravasation. Effective volume loss and tissue gap effusion are the causes of OF in AP. Therefore, PDW could be a useful predictor of AP of POF.

POF, the main cause of death in AP, develops in 10%–20% of AP patients within the onset two weeks of disease, with a mortality rate between 20% and 50% [19, 20]. So the ability to predict and assess POF in AP patients in the early stage is quite important. Clinical history and physical exam with ultrasonography, computed tomography, and magnetic resonance imaging have been shown to contribute to diagnostic accuracy in patients with suspected SAP, but it is not effective all the time. There has been much effort to search for biomarkers to identify patients at risk for POF. However, most of them are expensive and unavailable. Zhang et al. suggest that BISAP score may be a valuable source for risk stratification and prognostic prediction in Chinese patients with AP compared with APACHE II and the Ranson scoring system. They found the AUC for severity predicted by BISAP was 0.793 (95% confidence interval (CI): 0.700–0.886), and the AUC for mortality predicted was 0.791 (95% CI: 0.593–0.989) [26]. And most of these scoring systems have the AUC for severity prediction between 0.6 and 0.8 in both the training and the validation cohorts on admission [27]. But these scoring systems are always too complex to calculate immediately in actual clinical situation.

In summary, this is the first time to evaluate PDW as a marker of incidence of POF in AP. Our finding suggests that PDW could be a potential predictive factor of POF in AP. However, there are still several limitations of the present research. First, as an observational study, the causality role of PDW and POF in AP requires to be investigated further in a prospective validation study. Second, the sample size of our study is not large enough to detect the differences between all other markers effectively. Multicenter prospective studies with large sample size are necessary in the future.

## Figures and Tables

**Table 1:** Basic characteristic of AP patients according to PDW level (cut-off: 16.45%).

	PDW ≤ 16.45%	PDW > 16.45%	*P* value
Number	85	50	
Age, years	49.09 ± 13.53	48.28 ± 16.46	0.756
Male gender	36 (42.3%)	33 (66.0%)	0.008
Daily drinker	30 (35.3%)	22 (44.0%)	0.319
Smoke	33 (38.3%)	25 (50.0%)	0.208
Etiology			0.001
Biliary	55 (64.7%)	17 (34.0%)	
Alcohol	15 (17.6%)	16 (32.0%)	
Hyperlipidemia	12 (14.1%)	12 (24.0%)	
Idiopathic	3 (3.5%)	5 (10.0%)	
Outcomes			
PNec	20 (23.5%)	26 (52.0%)	0.001
POF	4 (4.7%)	26 (52.0%)	<0.001
In-hospital mortality	4 (4.7%)	7 (14.0%)	<0.001

AP: acute pancreatitis; PDW: platelet distribution width; PNec: pancreatic necrosis; POF: persistent organ failure.

**Table 2:** Types of POF and the corresponding pancreatic necrosis.

	POF	PNec
Solitary POF	26	22 (81.5%)
Respiratory	26 (100.0%)	22 (81.5%)
Renal	0 (0.0%)	0 (0.0%)
Cardiovascular	0 (0.0%)	0 (0.0%)
Multiple POF	4	4 (100.0%)
Respiratory + renal	4 (100.0%)	4 (100.0%)
Respiratory + cardiovascular	0 (0.0%)	0 (0.0%)
Respiratory + cardiovascular + renal	0 (0.0%)	0 (0.0%)

POF: persistent organ failure; PNec: pancreatic necrosis.

**Table 3:** Demographic and clinical characteristics of the patients with and without POF.

	All patients	Non-POF	POF	*P* value
Number	135	105	30	
Age, years	48.55 ± 14.83	49.4 ± 14.01	46.7 ± 16.70	0.369
Male gender	69 (51.1%)	54 (51.4%)	15 (50.0%)	0.891
Daily drinker	52 (38.5%)	39 (37.1%)	13 (43.3%)	0.542
Current smoker	58 (43.0%)	45 (42.9%)	13 (43.3%)	0.963
Etiology				0.121
Biliary	72 (53.3%)	62 (59.0%)	10 (33.3%)	
Alcohol	31 (29.5%)	20 (19.0%)	11 (36.7%)	
Hyperlipidemia	24 (22.9%)	16 (15.2%)	8 (26.7%)	
Idiopathic	8 (5.9%)	7 (6.7%)	1 (3.3%)	

POF: persistent organ failure.

**Table 4:** Laboratory and clinical data of the patients with and without POF.

Laboratory data				
Hemoglobin, /L	142.07 ± 27.54	137.86 ± 26.18	156.8026.57	0.001
Platelet count, ×10^9^/L	199.61 ± 61.49	202.98 ± 57.85	187.80 ± 72.68	0.234
Serum glucose, mmol/L	8.90 ± 4.15	7.80 ± 2.79	12.76 ± 5.65	<0.001
Aspartate aminotransferase, U/L	96.52 ± 144.83	101.46 ± 157.22	79.23 ± 88.80	0.461
Albumin, g/L	36.43 ± 5.57	37.53 ± 4.95	32.56 ± 5.95	<0.001
Serum calcium, mmol/L	2.04 ± 0.29	2.13 ± 0.21	1.71 ± 0.31	<0.001
Platelet distribution width, %	15.48 ± 2.46	14.88 ± 2.24	17.60 ± 1.96	<0.001
Severity scores				
^∗^Ranson score	3.68 ± 1.86	3.37 ± 1.87	4.77 ± 1.41	<0.001
^∗^SIRS score	2.30 ± 1.02	2.13 ± 1.01	2.90 ± 0.84	<0.001
Outcomes				
PNec	46 (34.1%)	21 (20.0%)	25 (83.3%)	<0.001
In-hospital mortality	11 (8.1%)	0 (0.0%)	11 (36.7%)	<0.001

POF: persistent organ failure; PNec: pancreatic necrosis. ^∗^Ranson scoring system: (1) age > 55 years; (2) serum glucose > 11.1 mmol/L; (3) aspartate aminotransferase > 250 U/L; (4) lactate dehydrogenase > 350 U/L; (5) white blood count > 16 × 10^9^/L; (6) hematocrit decrease > 10%; (7) serum urea increase > 1 mmol/L; (8) PaO_2_ < 60 mmHg; (9) serum calcium < 2 mmol/L; (10) base deficiency > 4 mmol/L; and (11) fluid loss > 6 L. Each event represents one point. ^∗^SIRS scoring system: (1) heart rate > 90 bpm; (2) temperature > 38°C or <36°C; (3) white blood count > 12 × 10^9^/L or < 4 × 10^9^/L; (4) breath rate > 20 bpm; and (5) PCO_2_ < 32.33 mmHg. Each event represents one point.

**Table 5:** Uni- and multivariable logistic regression analyses of risk factors for POF.

	Univariable analysis		Multivariable analysis	
	Odd ratio (95% CI)	*P* value	Odd ratio (95% CI)	*P* value
Sex, male	0.94 (0.42, 2.13)	0.89		
Age, ≥60 years	1.12 (0.46, 2.74)	0.8		
^∗^Hemoglobin, >150 g/L	2.98 (1.30, 6.86)	0.01		
^#^Glucose ≥ 11.1 mmol/L	7.85 (3.17, 19.41)	<0.001		
Albumin < 32 g/L	4.72 (1.86, 11.99)	0.001	7.18 (1.73, 29.79)	0.007
^#^Calcium < 2 mmol/L	26.00 (8.18, 82.62)	<0.001		
PDW ≥ 16.45%	21.94 (6.97, 69.07)	<0.001	39.42 (8.64, 179.77)	<0.001
Ranson score ≥ 4	4.75 (1.79, 12.57)	0.002	2.38 (0.58, 9.76)	0.227
SIRS score ≥ 3	5.743 (2.32,14.22)	<0.001	3.78 (0.99,14.36)	0.051

^#^As serum glucose and serum calcium were not independent of Ranson score, they were excluded from multivariable analysis. ^∗^Due to the small number of the study population and because the OR of albumin is higher than that of hemoglobin in the univariable model, we decide to include albumin, PDW, and Ranson score in multivariable analysis. CI: confidence interval; PDW: platelet distribution width.

**Table 6:** Receiving operator curve analysis in diagnosing POF.

	AUC (95% CI)	Cut-off	Sensitivity	Specificity	PPV	NPV
Hemoglobin (g/L)	0.694 (0.576–0.812)	150	0.533	0.695	0.333	0.845
Platelet count (×10^9^/L)	0.601 (0.478–0.724)	100	0.067	0.971	0.400	0.785
Serum glucose (mmol/L)	0.794 (0.697–0.891)	11.1	0.567	0.857	0.545	0.882
Albumin (g/L)	0.726 (0.628–0.823)	32	0.400	0.876	0.435	0.821
Serum calcium (mmol/L)	0.888 (0.829–0.948)	2.0	0.867	0.790	0.553	0.954
PDW (%)	0.870 (0.801–0.939)	16.45	0.867	0.771	0.520	0.953
Ranson score	0.727 (0.638–0.816)	4	0.717	0.372	0.333	0.905
SIRS score	0.608 (0.500–0.716)	3	0.833	0.329	0.393	0.898

AUC: area under the curve; CI: confidence intervals; PDW: platelet distribution width; POF: persistent organ failure; ROC: receiver operating curve; PPV: positive predictive value; NPV: negative predictive value.
